# Co-reactant-free self-enhanced solid-state electrochemiluminescence platform based on polyluminol-gold nanocomposite for signal-on detection of mercury ion

**DOI:** 10.1038/s41598-021-86195-1

**Published:** 2021-03-25

**Authors:** Chikkili Venkateswara Raju, Shanmugam Senthil Kumar

**Affiliations:** 1 Electrodics and Electrocatalysis Division, CSIR-Central Electrochemical Research Institute (CECRI), Karaikudi, Tamil Nadu 630003 India; 2 Academy of Scientific and Innovative Research (AcSIR), Ghaziabad, Uttar Pradesh 201002 India

**Keywords:** Analytical chemistry, Electrochemistry

## Abstract

Development of a self-enhanced solid-state ECL platform creates a straightforward experimental design for the fabrication of point-of-care applications. Herein, we develop a promising method for self-enhanced solid-state ECL platform of polyluminol gold nanocomposite on glassy carbon electrode [(PL-Au)_nano_/GCE] via simple one-step electrochemical deposition process without involving any additional co-reactants. The presence of gold nanoparticles (AuNPs) augments the electron transfer kinetics of PL (polyluminol) and enhances the solid-state ECL intensity and promotes label-free, excellent sensitivity, and selectivity to detect Hg^2+^ in physiological pH through signal-on mode. Unlike pristine PL/GCE, electrochemically co-deposited AuNPs in the (PL-Au)_nano_/GCE composite, enable the co-reactant accelerator by improving the catalytic activity of PL towards oxygen reduction reaction (ORR) yielding in-situ ROS (co-reactant) generation. Further, the ECL intensity of (PL-Au)_nano_/GCE composite, gradually increases with each addition of Hg^2+^ ion. This is because of the formation of an amalgamation of Au-Hg on (PL-Au)_nano_/GCE composite surface which further accelerates the yield of in-situ ROS and enhances the intensity of ECL. Whereas no ECL signals changes were observed for PL/GCE composite. The proposed self-enhanced solid-state ECL platform is selectively sensing the Hg^2+^ ion in the linear range of 0.3–200 nM with a detection limit of 0.1 nM. The demonstrated (PL-Au)_nano_/GCE platform might pave new avenues for further studies in the solid-state ECL platform which could be more useful in on-site monitoring of clinical bioassay and immunosensors.

## Introduction

Electrogenerated chemiluminescence (ECL) is a process of light emission which is due to an energetic interaction between electrogenerated species on electrode and electrolyte interface^1^. ECL has a great advantage over chemiluminescence (CL) because of low background signals, simple optical setup, good spatial–temporal control, and versatility^2,3^. Recently, ECL becomes a popular and widely used analytical technique in clinical diagnostics, material science, and environmental monitoring^4,5^. Even though Ru(bpy)_3_^2+^ based ECL systems such as Ru(bpy)_3_^2+^/tri-n-propylamine (TPrA)^6^, Ru(bpy)_3_^2+^-oxalate (C_2_O_4_^2−^)^7^ and Ru(bpy)_3_^2+^-hydrogen peroxide (H_2_O_2_)^8,9^ are more acitve ECL systems in reported litrature, still luminol-H_2_O_2_^10^ based ECL systems have more attractive glance because of its non-toxicity, cost-efficient, and high quantum yield with low oxidation potential^11,12^. The luminescent property of the luminol-H_2_O_2_ system mostly depends on its inherent chemical or electrochemical reaction through the intermediates of oxygen species like OH^**∙**^, OH^−^, and O_2_^−^^13,14^. The major limitation of luminol’s ECL property is hampered by its poor aqueous solubility, alkaline mediated ECL dependency. To overcome this, water-soluble luminol derivatives were chemically synthesized with enhanced ECL intensity in an aqueous electrolyte^15^. However, the adopted synthesis involves multiple steps, desires tedious reactions without scalability. Moreover, the co-reactant like H_2_O_2_ is also not quite stable at room temperature which also suppresses the efficiency of the ECL signal of luminol^16^. One can overcome this problem by generating the reactive oxygen species (ROS) by using co-reactant accelerators which can produce more ROS through the reduction of dissolved oxygen and enhances the stability of the ECL signal^17^. In ECL, the co-reaction or co-reactant accelerators are playing a crucial role in boosting the ECL intensity of luminophore by dissociating the co-reactant into active radicals^18^. For example, the ECL intensity of luminol in O_2_ saturated electrolyte increased by 2-fold by the co-reactant accelerator strategy^19^.

Because of mass transport limitation, a homogeneous phase or solution based luminol system always inhibits the efficiency of ECL emission, impeding the low molecular detection of selected analytes^20–22^. Use of heterogeneous or solid-state ECL has several advantages such as a minimum amount of luminophores is sufficient, simplifies the experimental setup, enhance more ECL intensity^23–25^. In this context, luminol is an aniline monomer derivative having an ECL inert –NH_2_ group facilitating the electro-polymerization in an acidic solution to form a stable PL film on the electrode surface^26^. However, the PL films have less electrical conductivity in neutral or alkaline solution, which needs further improvisation for highly efficient luminol system. Despite few attempts were taken in functionalizing the luminol system in solution state with metal NPs, till now no efforts were taken to hybridize PL films particularly at solid-state, which eventually have potential scope for point-of-care/on-site application.

Mercury ion (Hg^2+^) is a heavy metal known for the individual as well as environmental toxicity^27^. The presence of Hg^2+^ in the human body causes brain damage and other chronic diseases^28,29^. Enzymes like horseradish peroxidase^30^, glucose oxidase^31^, invertase^32^ and urease^33^ have a strong affinity with Hg^2+^ which inhibits the functions of the enzymes of the human body. Therefore, the sensitive and selective method requires in detecting Hg^2+^ at a lower level is highly beneficial for environmental application as well as healthcare. Though spectroscopic and chromatographic techniques are well documented as well as in practice for detection of Hg^2+^, nevertheless modern analytical methods demand highly sensitive yet selective portable assay platform. Even though luminol based ECL property is established for Hg^2+^ estimation, still it requires specific bio-receptors like DNA^34^ and enzymes^35^ for selective ECL quenching. Thus, there is a potential need for the development of a label-free solid-state ECL platform beneficial for selective detection of Hg^2+^ ions.

Herein, a simple one-step electrochemical strategy is established for the preparation of (PL-Au)_nano_/GCE which displayed the self-enhanced solid-state ECL signal, thereby enabling label-free detection of Hg^2+^ via signal-on ECL mode. Incorporation of electrochemically co-deposited AuNPs with PL films acts as a co-reactant accelerator to enhancing the ECL intensity by producing more ROS. The optimized condition is selective for Hg^2+^ detection without influence from other metal ions. The obtained results were superior/specific to Hg^2+^ ion over the pristine PL/GCE and other noble metal composites (PL-Pt)_nano_/GCE and (PL-Ag)_nano_/GCE. Hence, the in-situ generated ROS is utilized as co-reactant, the proposed methodology does not require any addition of co-reactant into electrolyte to study the ECL of PL.

## Experimental section

### Chemicals

Luminol (97%), Disodium hydrogen phosphate (Na_2_HPO_4_∙7H_2_O), Sodium dihydrogen phosphate mono hydrate (NaH_2_PO_4_∙H_2_O), Tetrachloroauric (III) acid (99.9%), Hexachloroplatinic (IV) acid hydrate (99.9%), Sulphuric acid (18.3 M), Mercury chloride (HgCl_2_), Silver nitrate (99.99%) and all other metal salts and benzoquinone were purchased from Alfa aesar. All the chemicals were used without any further purification. O_2_ gas saturated electrolyte solution in electrochemical cell is achieved by continuous purging of O_2_ (purity of 99.99%) gas into the electrolyte solutions (0.1 M PBS) using typical design of four neck electrochemical cell with gas purging setup using capillary serves as inlet tube that inserted into the electrolytic cell which is directly connected to O_2_ gas cylinder. O_2_ gas was purged into the electrolyte solution for 45 min to complete saturation. The similar method used to saturate the electrolyte with argon gas (Ar) of 99.99% purity. Milli-Q water (18.2Ω) used as a solvent for preparing the electrolyte solution. 0.5 M H_2_SO_4_ stock solution was prepared by diluting 18.3 M H_2_SO_4_.

### Electrochemistry and ECL measurements

Commercially available glassy carbon electrode (GCE) with 0.07065 cm^2^ surface area serves as working electrode, platinum foil as the counter electrode and Ag/AgCl is used as reference electrode respectively. Cyclic voltammetry (CV) and potential step experiments were performed with an Autolab electrochemical workstation (EcoChemie, The Netherlands). The ECL along with CV signals is measured simultaneously with a photomultiplier tube (PMT, Hamamatsu H9305-04). The PMT was held at − 500 V with a high-voltage power supply. The photo current generated at the PMT was converted to a voltage using an electrometer system (model 6517, Keithley, Cleveland, OH) and connected to the Autolab via an analog-to-digital converter (ADC).

### Preparation of PL/GCE and (PL-Au)_nano_/GCE

**Figure 1:**
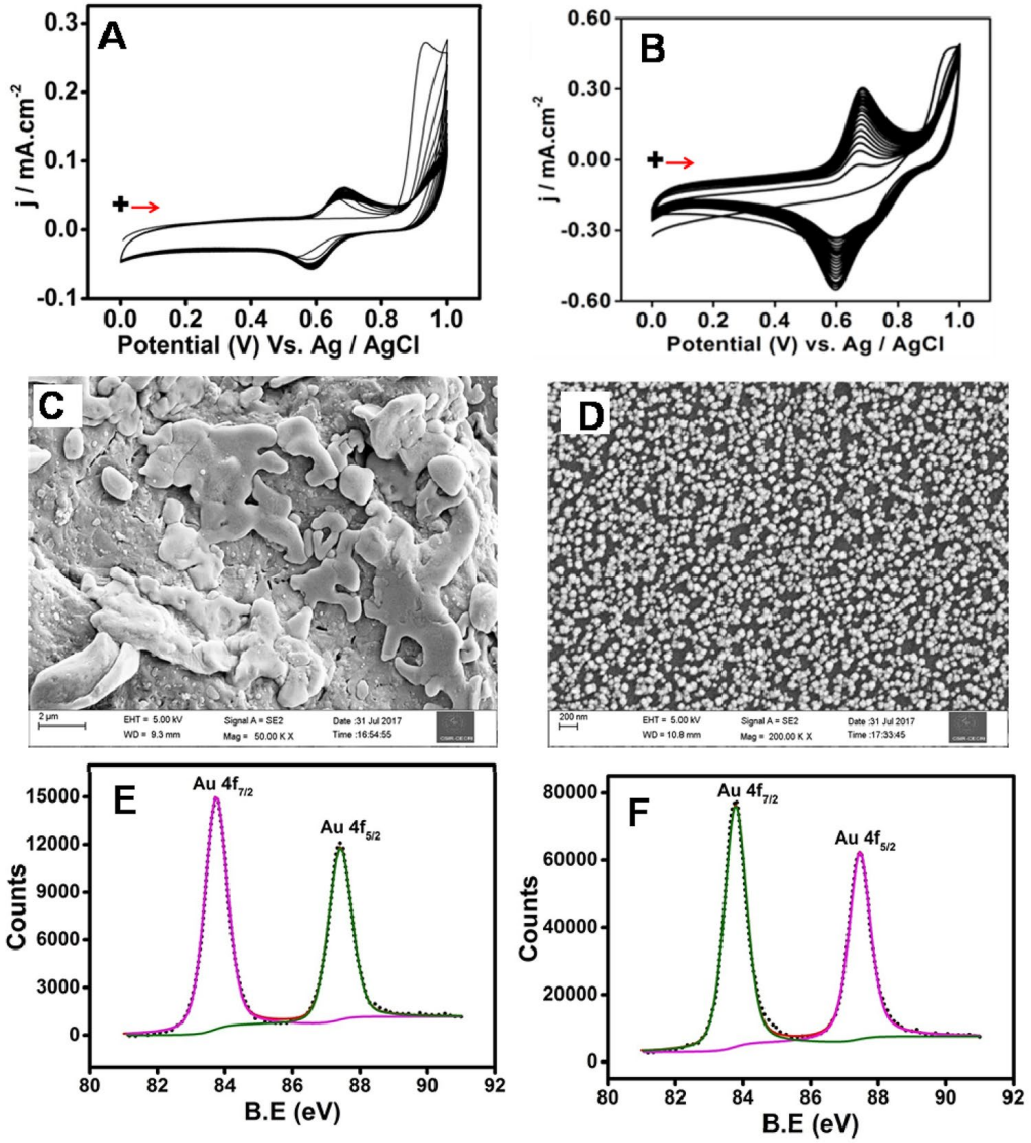
Repetitive CVs of 1 mM luminol (**A**) and 1 mM luminol + 1.5 mM HAuCl_4_ (**B**) in 0.5 M H_2_SO_4_ at the scan rate of 0.1 V/s. FESEM images of PL/GCE (**C**) and (PL-Au)_nano_/GCE (**D**). The XPS spectrum (4f_5/2_ and 4f_7/2_ of Au) of (PL-Au)_nano_/GCE before and after etching (**E**,**F**) respectively.

**Scheme 1:**
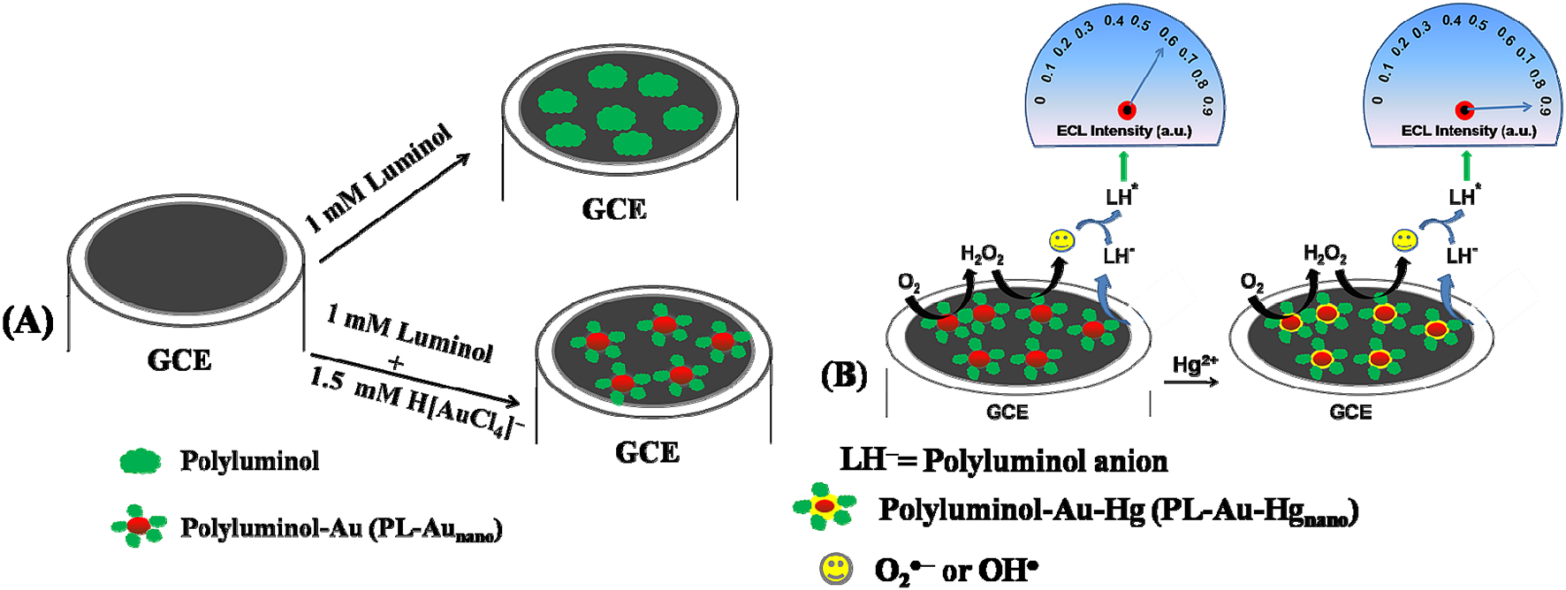
Schematic illustration of PL/GCE and (PL-Au)_nano_/GCE preparation (**A**) and ECL mechanism of (PL-Au)_nano_/GCE before and after Hg^2+^ addition (**B**).

PL-Au nano-composite was electrochemically deposited on GCE as follows. Initially, GCE was successively polished with Al_2_O_3_ slurry (0.3, 0.05 µm) then sonicated for 5 min in an ultrasonic bath with distilled water at room temperature. Further GCE was electrochemically cleaned in 0.5 M H_2_SO_4_ by cycling at 0.1 V/s from 0 to 1.2 V vs. Ag/ AgCl for 10 cycles. After that, polished GCE was immersed in an electrochemical cell containing 1 mM luminol in 0.5 M H_2_SO_4_ and electrochemically treated about 20 cycles in the range of 0 to 1 V at the scan rate of 0.1 V/s (Fig. 1A). After the electrochemical treatment, the electrode was washed thoroughly with milli-Q water, the modified electrode was termed to as PL/GCE. Similar way (PL-Au)_nano_/GCE prepared by taking 1 mM luminol + 1.5 mM HAuCl_4_ in 0.5 M H_2_SO_4_ (Fig. 1B). The electrode modification process is also represented in the Scheme 1A. The similar procedure was followed to deposit (PL-Pt)_nano_ and (PL-Ag)_nano_ on GCE using 1 mM luminol + 1.5 mM PtCl_4_^2−^ and 1 mM luminol + 1.5 mM AgNO_3_ respectively, in 0.5 M H_2_SO_4_ which can be referred as (PL-Pt)_nano_/GCE and (PL-Ag)_nano_/GCE.

### Characterization techniques

We used Field emission scanning electron microscope (FESEM, Supra 55 VP, Carl Zeiss), Energy dispersive X-ray (EDX, Oxford Instruments X-MAX, 20 mm^2^) analysis, and Atomic force microscope (AFM, Agilent technologies 5500 instruments) technique in order to know the morphological changes and elemental presence after modification of GCE. The X-ray photoelectron spectroscopic (XPS) technique used to predict the state of elements present in the outermost part of composite by using Theta Probe AR-XPS System, Thermo Fisher Scientific (UK).

### ECL spectrum

ECL spectrum is recorded using the optimized PMT voltage as 950 V and slit width is 20 nm in spectrofluorimeter. Also, applied a constant potential pulse at 0.6 V vs Ag/AgCl on (PL-Au)_nano_/GC plate (1 × 1 cm^2^) in O_2_ saturated 0.1 M PBS at pH 7.4.

## Results and discussions

### Electrochemical studies of PL/GCE and (PL-Au)_nano_/GCE

Figure 1A,B depicts the cyclic voltammetry of PL and (PL-Au)_nano_ growth patterns on a glassy carbon substrate. As seen in Fig. 1A, a sharp increase in peak current at 0.9 V for the 1st cycle of CV which is due to the luminol oxidation where the radical polymerization of luminol starts and decreased in the peak current (at 0.9 V) during the subsequent electrochemical cycling. Moreover, there is a reversible redox peak growth which is due to the reduction (E_Pc_ = 0.6 V) and oxidation (E_pa_ = 0.7 V) of PL. The redox peaks current is gradually increases for initial few cycles (up to 10 cycles) and then exhibit a stable redox response without further increase in redox peak current. Interestingly, the existence of HAuCl_4_ with luminol causes incremental redox peak current density of PL during electrochemical cycling without change in inherent redox potential. In addition, a small reduction peak at 0.8 V observed which is due to the reduction of Au^3+^ to Au^0^ (Fig. 1B). To ensure this particular reduction reaction a control experiment with pristine HAuCl_4_ was performed. To this only HAuCl_4_ is dissolved in 0.5 M H_2_SO_4_ without luminol yield at sharp reduction peak at exactly 0.8 V and decreased upon continuous electrochemical cycling (Supplementary Fig. S1A). After electrochemical cycling, the typical redox response of modified GCE (AuNPs/GCE) in 0.5 M H_2_SO_4_ confirms the formation of AuNPs on GCE (Supplementary Fig. S1B). The CV of typical stable redox response of PL/GCE and (PL-Au)_nano_/GCE clearly seen in the potential window of 0–1 V in 0.5 M H_2_SO_4_ solution (Supplementary Fig. S2A,B), evidencing the deposition of PL^36^. However, the sweeping potential region is extended up to 1.7 V (Supplementary Fig. S3) to validate the existence of typical redox characteristics of AuNPs in line with redox peaks of PL (at 0.7 V and 0.6 V). From the second cycle onwards the redox peak of PL is started to disappear with retention of AuNPs characteristic redox behavior, suggesting an over oxidation potential resulted from either leaching or deactivating the PL in 0.5 M H_2_SO_4_. And also, the calculated AuNPs reduction peak charge in AuNPs/GCE (Supplementary Fig. S1B) is almost 2-fold less when compared with the reduction peak charge of AuNPs in the (PL-Au)_nano_/GCE (Supplementary Fig. S3). Moreover, the peak current and charge associated with oxidation (at 0.7 V) and reduction (at 0.6 V) peak of PL in (PL-Au)_nano_/GCE is also higher than that of pristine PL/GCE (Supplementary Table S1). This is because of the simultaneous growth deposition of AuNPs and PL film on GCE.

The surface morphology of electrochemically deposited PL/GCE and (PL-Au)_nano_/GCE was studied by using FE-SEM (Fig. 1C,D). PL/GCE shows microstructures of polymer islands (Fig. 1C), on the other hand (PL-Au)_nano_/GCE exhibit homogenous spherical nanostructures with an average size distribution of ~ 60 nm (Fig. 1D), evidencing the existence of nanocomposite on the GCE. To elucidate the elemental compositions and surface chemistry of the nanocomposite on GCE surface an XPS study was performed. Survey spectra of the prepared platform were presented in Supplementary Fig. S4. The high-resolution XPS spectra of Au 4f_7/2_ and Au 4f_5/2_ is denoted in Fig. 1E. The observed binding energies are comparable with the standard values of pure gold viz., 83.8 eV (CAS No.7440-57-5) and 87.43 eV (CAS No.7440-57-5) corresponded to 4f_7/2_ and 4f_5/2_, respectively. In order to understand the localization pattern of Au and PL films an in-situ etching was performed using XPS analysis. Figure 1F illustrates the XPS spectrum of etched (PL-Au)_nano_/GCE with an amplified signal intensity of Au 4f_7/2_ and 4f_5/2,_ revealing enhanced exposure of Au via stripping of PL. This was further supported by the existence of weaker O1s and N1s peaks (Supplementary Fig. S5).

To complement the surface topography and elemental structure an AFM and EDX spectral measurements were performed and the results are presented in Supplementary Fig. S6. The EDX of PL/GCE is showing C, N, and O elements (Supplementary Fig. S6A) whereas Au along with C and O presents in (PL-Au)_nano_/GCE composite (Supplementary Fig. S6B). This result was evidencing that PL and AuNPs were co-deposited on the GCE surface. In addition, the AFM analysis is also displayed macrostructure kind of morphology for PL/GCE (Supplementary Fig. S6C), but nanostructured with spherical shapes presents in (PL-Au)_nano_/GCE composite (Supplementary Fig. S6D) as similar with FE-SEM. Electrical conductivity and charge transfer kinetics for the prepared (PL-Au)_nano_/GCE was measured using electrochemical impedance spectroscopy (EIS). The calculated charge transfer resistance (∆R_ct_) derived from the Nyquist plot of PL/GCE and (PL-Au)_nano_/GCE is 4076 Ω and 276 Ω, respectively (Supplementary Fig. S7). The lesser value of R_ct_ clearly confirms that the (PL-Au)_nano_/GCE exhibit better electrical conductivity and charge transfer kinetic than the pristine PL/GCE.

### ECL experiments and ECL mechanism

**Figure 2:**
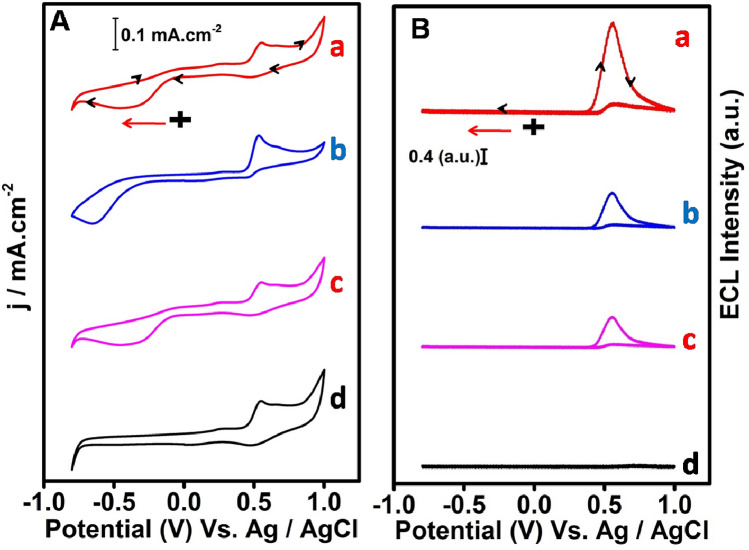
Simultaneously obtained CV (**A**) and its corresponding ECL responses (**B**) of (PL-Au)_nano_/GCE (a), PL/GCE (b), PL/pc-Au (c), in O_2_ saturated and (PL-Au)_nano_/GCE (d) in Argon saturated 0.1 M PBS (pH 7.4) at 0.1 V/s.

Unlike the redox response of (PL-Au)_nano_/GCE in 0.5 M H_2_SO_4_ (Supplementary Fig. S2A,B), the simultaneously recorded CV showed an irreversible peak at − 0.4 V and 0.6 V which is due to the dissolved oxygen reduction reaction (ORR)^37^ and PL oxidation peak (Fig. 2A,a) in O_2_ gas saturated 0.1 M PBS (pH 7.4) during scanning from 0 to − 0.8 V to + 1 V. In contrast, the pristine PL/GCE showed oxidation peak at 0.6 V but the oxygen reduction peak observed at high cathode potential of − 0.65 V (Fig. 2A,b). The less cathode peak potential shift approximately − 0.25 V and high reduction current density for dissolved O_2_ reduction clearly indicates that the (PL-Au)_nano_/GCE is highly catalytic than pristine PL/GCE. To understand the role of AuNPs, the PL film also deposited on a polycrystalline gold surface (PL/pc-Au) which exhibits the redox response in O_2_ gas saturated 0.1 M PBS (pH 7.4) (Fig. 2A,c). Although the peak potentials of PL oxidation and ORR are similar to the (PL-Au)_nano_/GCE, the peak current density of PL oxidation and ORR is quite decreased. This further confirms the (PL-Au)_nano_/GCE shows superior electrocatalytic behavior towards ORR. The simultaneous record of ECL from these modified electrodes shown in Fig. 2B.

**Figure 3:**
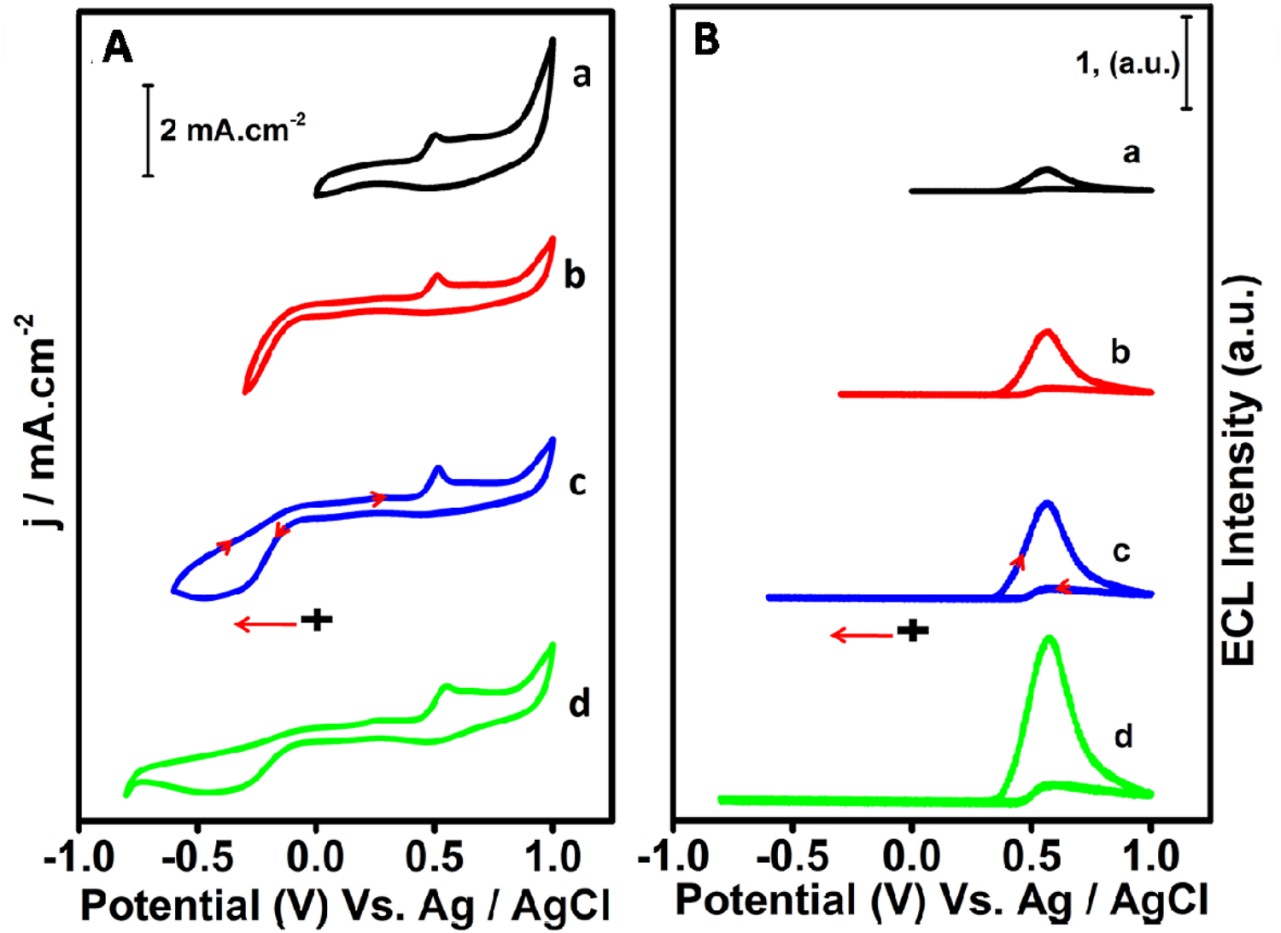
Cyclic voltammograms (**A**) and corresponding ECL signals (**B**) of (PL-Au)_nano_/GCE in O_2_ saturated 0.1 M PBS (pH 7.4) at several scanned potentials, (a) 0 to 1 V, (b) − 0.3 to 1 V, (c) − 0.6 to 1 V and (d) − 0.8 to 1 V.

As expected, a high intense ECL peak was observed at 0.6 V where exactly an oxidation of PL peak exists (Fig. 2A,a) in (PL-Au)_nano_/GCE. The relative intensity of the observed ECL signal of (PL-Au)_nano_/GCE is almost three times higher than PL/GCE (Fig. 2B,b) and PL/pc-Au (Fig. 2B,c) composites. Under completely argon gas saturated 0.1 M PBS, the (PL-Au)_nano_/GCE shows the disappearance of ORR and ECL peak (Fig. 2A,d,B,d). All these results clearly confirm that, obtained ECL signals are totally dependent on the concentration of dissolved O_2_ present in the electrolyte solution. The in-situ generated reactive oxygen species (ROS) from dissolved O_2_ act as co-reactant which eventually react with PL anion to produce ECL signal. The ECL enhancement of (PL-Au)_nano_/GCE is perhaps due to the presence of AuNPs which behave as a co-reactant accelerator by producing the ROS radicals via ORR. In order to understand the significance of AuNPs on PL film, the same PL film electrodeposited in the presence of H_2_PtCl_4_ and AgNO_3_ with in the same experimental conditions of (PL-Au)_nano_/GCE (Supplementary Fig. S8A,B). Interestingly, there is no ECL observed for (PL-pt)_nano_/GCE and (PL-Ag)_nano_/GCE composites (Supplementary Fig. S8C,D). Owing to its well-known catalytic behavior of Pt in ORR, the reduction of O_2_ to H_2_O occurs via a direct 4e^−^ pathway and it may not follow the 2e^−^ pathway which is necessary for H_2_O_2_ and ROS generation^38,39^. Even though luminol and Ag have good interaction and enables excellent chemiluminescence activity in presence of H_2_O_2_^40^. The present experimental conditions do not show any ECL for (PL-Ag)_nano_/GCE suggesting (PL-Ag)_nano_/GCE film could not act as ROS generator in solid-state ECL platform. Thus, the only AuNPs present in (PL-Au)_nano_/GCE has effective co-competence of a co-reactant accelerator to generate more ROS thereby promoting the ECL activity. Further, the effect of potential window on ECL intensity was studied by varying the initial potentials. Figure 3A,B shows the CV and corresponding ECL responses of (PL-Au)_nano_/GCE composite at various scan directions such as 0 to 1 V, − 0.3 to 1 V, − 0.6 to 1 V, and − 0.8 to 1 V respectively in O_2_ saturated 0.1 M PBS (pH 7.4). As illustrated in Fig. 3B, ECL intensities of (PL-Au)_nano_/GCE composite vary on change of potential scan direction. We observed a high intense ECL signal for − 0.8 to 1 V (Fig. 3B,d) scans direction, because at this particular direction maximum amount of ROS generated by the reduction of O_2_ (Fig. 3A,d). A small intense ECL signal is observed when we scan 0–1 V. Overall, the ECL intensity of (PL-Au)_nano_/GCE composite gradually increases by changing the potential scan towards a more negative to positive direction and concluded that ECL of (PL-Au)_nano_/GCE composite depends on the in-situ generated ROS. The ROS further oxidizes during anodic direction then reacts with poly luminol anion to emit light. The charge associated with ORR peak of Fig. 3A,b–d is calculated by integrating the peak area and the value is given in Supplementary Table S2. The maximum charge is obtained for the ORR peak of − 0.8 to 1 V scan direction, which is an evidence for more amounts of ROS generated at this potential scan.

To validate the ECL experimental conditions and its associated effect of ECL intensity of (PL-Au)_nano_/GCE, the different concentrations of luminol and HAuCl_4_.3H_2_O were taken for electro-deposition (see in Supplementary Sect. 2). From the experimental observation it is found that 1.5 mM of HAuCl_4_·3H_2_O and 1 mM luminol is sufficient for deposition of (PL-Au)_nano_/GCE composite, which apparently enabled a high intense ECL signal (Supplementary Figs. S9, S10). The reproducibility test was carried out by repeating the ECL experiments of (PL-Au)_nano_/GCE composite by four different time intervals in O_2_ saturated 0.1 M PBS (pH 7.4) at 0.05 V/s. The ECL intensity at different repetitions is shown as bar chart diagram (Supplementary Fig. S11), the ECL intensity was almost const at each repeated experiments.

**Figure 4:**
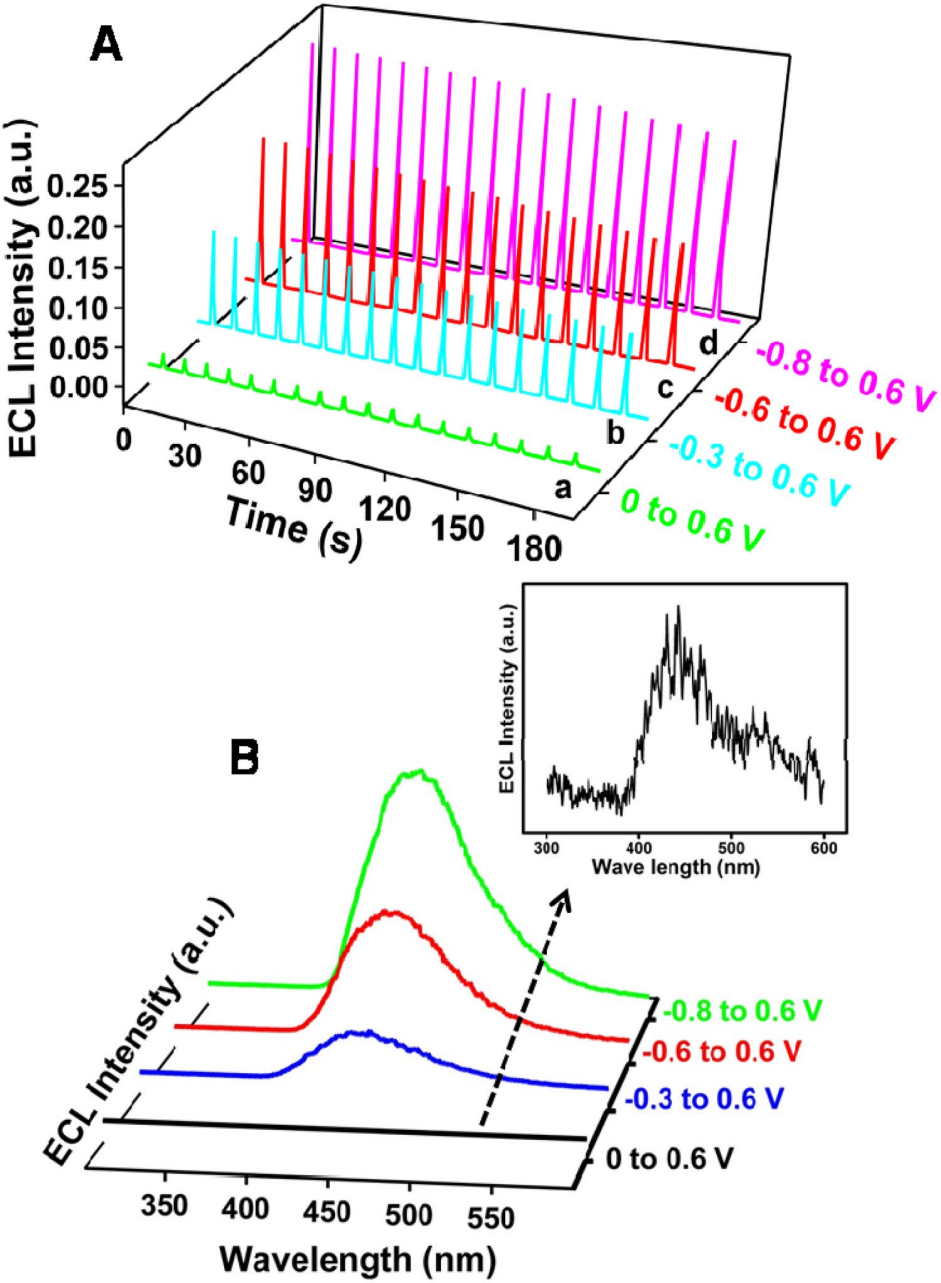
ECL intensity vs. time curves of (PL-Au)_nano_/GCE (**A**) and ECL spectrum (**B**) in O_2_ saturated 0.1 M PBS (pH 7.4) at several pulse potentials such as 0 to 0.6 V (a), − 0.3 to 0.6 V (b), − 0.6 to 0.6 V (c) and − 0.8 to 0.6 V (d).

Since the ECL signal of (PL-Au)_nano_/GCE composite depends on the in-situ generated ROS, we performed a time based-ECL transient experiments by changing the initial stepping potentials to study the ECL stability of ROS in O_2_ saturated 0.1 M PBS (pH 7.4). Figure 4A shows the ECL intensity *vs.* time transients of (PL-Au)_nano_/GCE composite at various initial potentials such as 0 (about 6 s) to 0.6 V (2.5 s), − 0.3 (6 s) to 0.6 V (2.5 s), − 0.6 (6 s) to 0.6 V (2.5 s) and − 0.8 (6 s) to 0.6 V (2.5 s) respectively in O_2_ saturated 0.1 M PBS (pH 7.4). The obtained ECL signals are quite stable even up to 18 consecutive cycles at various initial step potentials. Among different initial potential pulse, the potential pulse between − 0.8 to 0.6 V, displays more intense and stable ECL signals which may be ascribed to the generation of more number of ROS at the interface of (PL-Au)_nano_/GCE (Fig. 4A).

The ECL spectrum of (PL-Au)_nano_/GCE (Fig. 4B) also performed to elucidate the wavelength of PL at various initial potentials in O_2_ saturated 0.1 M PBS (pH 7.4). As seen from Fig. 4B, ECL signals were observed only at 430 nm which is consistent with the photoluminescence spectrum. Further, the highest intensity of ECL spectrum is obtained for the pulse potential of − 0.8 to 0.6 V, indicating the highest ROS generation in the specified potentials. This again complements the consistent of ECL transient experiment results (vide supra). Further to prove the concept that ROS playing a crucial role in enhancing the ECL intensity of (PL-Au)_nano_/GCE, we studied ECL experiments in the presence of ROS scavengers such as superoxide dismutase (SOD) and benzoquinone (BQ)^17,41^. It is worthy to note that OH^∙^ and O_2_^∙−^ selectively quenched by SOD and BQ respectively (Supplementary Fig. S12A,B) which confirm the ECL emission is due to formation of ROS radicals (OH^∙^ and O_2_^−^). Based on all the above results, the ECL reaction mechanism of (PL-Au)_nano_/GCE in the presence of dissolved O_2_ is as follows.

1 $$ {\text{O}}_{{2}} + {\text{ 2e}}^{ - } \to {\text{H}}_{{2}} {\text{O}}_{{2}} $$

2 $$ {\text{H}}_{{2}} {\text{O}}_{{2}} \to {\text{O}}_{{2}}^{ \cdot - } /{\text{ OH}}^{ \cdot } $$

3 $$ \left( {{\text{PL}} - {\text{Au}}} \right) \, - {\text{ e}}^{ - } \to ({\text{PL}}^{ \cdot - } - {\text{Au}}) \, + {\text{ H}}^{ + } $$

4 $$ ({\text{PL}}^{ \cdot - } - {\text{Au}}) \, + {\text{ O}}_{{2}}^{ \cdot - } /{\text{ OH}}^{ \cdot } \to ({\text{PLO}}_{{2}}^{{{2} - }} - {\text{Au}}) $$

5 $$ ({\text{PLO}}_{{2}}^{{{2} - }} - {\text{Au}}) \to {\text{AP}}_{{2}}^{{{2} - *}} + {\text{ N}}_{{2}} $$

6 $$ {\text{AP}}_{{2}}^{{{2} - *}} \to {\text{AP}}_{{2}}^{{{2} - }} + {\text{ h}}\nu $$

### Determination of Hg^2+^ ion

It has been identified in the literature that, the AuNPs and Hg^2+^ ion has strong specific metallophilic interaction and form spontaneous Au-Hg amalgam which tunes the catalytic properties of AuNPs and accelerate the rate of decomposition of H_2_O_2_^42^. Such study can be escalated for various applications particularly in environmental pollutant monitoring, and detection of Hg^2+^ as preservatives in vaccines. Inspired from that herein, the effect of Hg^2+^ ion with (PL-Au)_nano_/GCE by exploring the various Hg^2+^ ion concentrations from 10 to 150 nM in O_2_ saturated 0.1 M PBS (pH 7.4) at 0.1 V/s (Supplementary Fig. S13A). Interestingly, the ECL intensity of (PL-Au)_nano_/GCE linearly increases with each addition of Hg^2+^ ion (Supplementary Fig. S13B) and reaches the maximum limit up to 150 nM, after that there is no much enhancement was observed. The observed enhancement of ECL intensity (signal on mechanism) during the addition of Hg^2+^ ion is due to the affinity between AuNPs and Hg^2+^ ion, leading to the formation of Au–Hg amalgam via aurophilic interactions^42^. As expected, the surface properties of AuNPs has been further changed upon interaction with Hg^2+^, and accelerate the in-situ generation of highly reactive ROS through catalytic reduction of dissolved oxygen^43^. The high ORR activity is a great sign in enhancing the ECL intensity of (PL-Au)_nano_/GCE and lead to detect the Hg^2+^ ion at trace level. The calibration curve of ECL intensity versus Hg^2+^ ion concentrations was represented in Supplementary Fig. S13B. A perfect linear relationship between ECL efficiency and Hg^2+^ concentrations were obtained from 10 to 150 nM. The detailed ECL mechanism of (PL-Au)_nano_/GCE before and after Hg^2+^ addition is shown in Scheme 1B.

**Figure 5:**
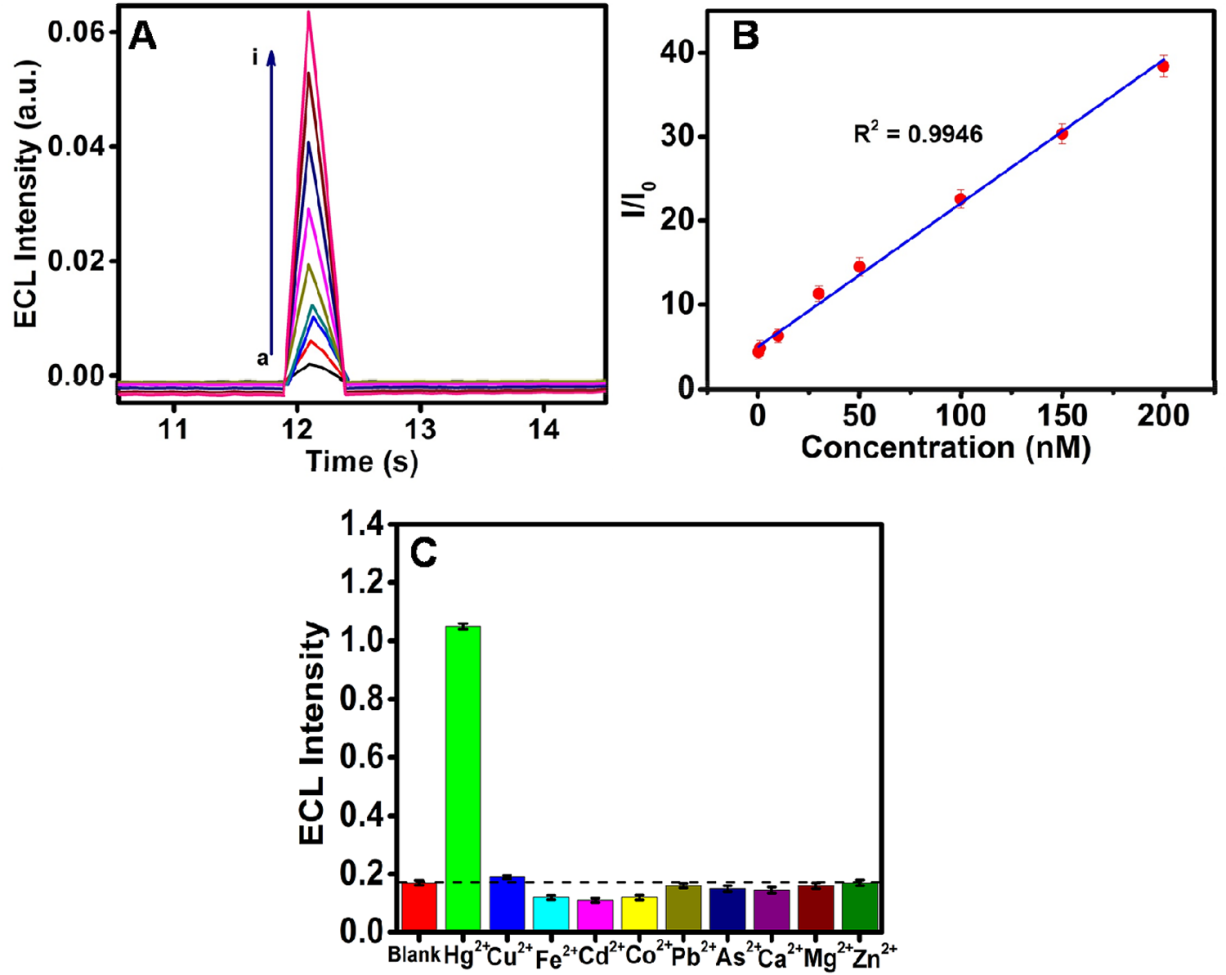
ECL intensity vs. time signals of ECL sensor (**A**) of (PL-Au)_nano_/GCE at various Hg^2+^ concentrations (a) 0, (b) 0.3, (c) 1, (d) 10, (e) 30, (f) 50, (g) 100, (h) 150 and (i) 200 nM by holding the potential of − 0.8 V for 10 s and 0.6 V holds about 0.5 s, its calibration curve (**B**), selectivity of ECL enhancement of (PL-Au)_nano_/GCE against some metal ions (**C**) in O_2_ saturated 0.1 M PBS (pH 7.4).

As seen in Scheme 1B, dissolved O_2_ get reduced on the electrode surface to produce highly reactive ROS. When the Hg^2+^ added into the electrolyte it was reduced and forms Au–Hg amalgam. Freshly formed Au–Hg amalgam further reduces the O_2_ to produces more number ROS which enhances the ECL intensity of (PL-Au)_nano_/GCE. Further, we performed a time vs. ECL intensity transient experiment to gain better sensitivity. Figure 5A depicts the obtained ECL transient curves at potentials pulse of holding the potential of − 0.8 V about 10 s and then 0.6 V held about 0.5 s in O_2_ saturated 0.1 M PBS (pH 7.4). The ECL intensity of (PL-Au)_nano_/GCE enhanced by each addition of Hg^2+^ ion in the linear range of 0.3–200 nM (Fig. 5B). The detection limit or limit of detection (LOD) is obtained by using 3× standard deviation/slope and the value is observed to be 0.1 nM and LOD is comparable with previous methods (Table 1). It is worthy to note that similar experiments were also performed by using PL/GCE in the presence of Hg^2+^ ion at different concentrations and there is no change in ECL intensity of PL/GCE during the Hg^2+^ addition (Supplementary Fig. S14). These results undoubtedly confirm that (PL-Au)_nano_/GCE composite only capable of producing more ECL in the presence of Hg^2+^ ion.

**Table 1:** Comparison of Hg^2+^ linear range and limit of detection with previous reported methods.

Methods	Probes	Linear range	LOD	References
ECL	DNA labelled with ruthenium complex	1 nM to 1 µM	0.3 nM	^44^
ECL	BSA protected Au–Ag bi-metallic clusters	10 nM to 5 µM	2.5 nM	^45^
ECL	Magnetic beads separation/collection process	1–250 nM	5 nM	^46^
ECL	Ru(phe)_3_^2+^/thymine on graphene oxide modified GCE	1 nM to 10 µM	0.34 nM	^47^
ECL	Ru(bpy)_3_^2+^doped silica nanoparticles	5 nM to 50 µM	2.3 nM	^48^
Fluorescence	Au NPs-DNA probe	80 nM to 6 µM	40 nM	^49^
Fluorescence	MnO_2_ nanosheet	0–20 nM	0.8 nM	^50^
ECL	(PL-Au)_nano_/GCE	0.3–200 nM	0.1 nM	In this work

### Interference study

In order to check the selectivity towards accelerator as well as detection of Hg^2+^ ion on (PL-Au)_nano_/GCE using ECL method, other metal ions such as Fe^2+^, Co^2+^, Pb^2+^, As^2+^, Cu^2+^, Cd^2+^, Zn^2+^ and Mg^2+^ were also added during the ECL measurements. The interference study has performed by using i–t transient experiment by holding the potential of − 0.8 V for 10 s and 0.6 V about 0.5 s. Figure 5C shows the bar diagram of ECL intensity with respect to 100 µM of all metal ions using (PL-Au)_nano_/GCE composite in O_2_ saturated 0.1 M PBS (pH 7.4). It can be notified that the high intense ECL signal is only in the case of Hg^2+^ addition, on the other hand, ECL signal from (PL-Au)_nano_/GCE against other studied metal ions were extremely low and comparable to the bare system. The obtained results further evidencing the selectivity of the prepared (PL-Au)_nano_/GCE system against the Hg^2+^, suggesting an ideal platform for real sample analysis.

### Real sample analysis

The Hg^2+^ ion in clinical samples like serum and environmental samples like tap water was tested by spiking the known concentrations. Initially, the tap water was boiled for few minutes to remove the contaminants, and then cooled at an open atmosphere until to reach room temperature. Afterward, the known concentrations of Hg^2+^ were spiked into the water and performed the ECL experiments. To detect the Hg^2+^ ion in serum samples a known amount of Hg^2+^ spiked and recorded the ECL experiments. The recovery of sample was also calculated in both tap water and serum indicated in Table 2.

**Table 2:** Hg^2+^ ion detection in tap water and serum samples.

Sample	Concentration		Recovery (% n = 3)
	Spiked (nM)	Found (nM)	
Tap water	30	28.3	94.3
	50	49.1	98.2
	70	69.3	99
	90	88.7	98.5
	100	98.9	98.9
Serum	30	29.4	98
	50	52.2	104.4
	70	68.9	98.4
	90	89.2	99.1
	100	99.6	99.6

From the observed results we can say that the proposed strategy to detect Hg^2+^ in real sample analysis could be possible. The percentage recoveries of the analyte were in the range of 93–104 suitable for practical application (Table 2). Further, we compared the Hg^2+^ recovery in tap water and serum samples by using Atomic absorption spectroscope (AAS) technique. The standard Hg^2+^ ion solutions of 25, 50, 100, 150, 250, 300, and 500 nM were calibrated, the linear plot shown in Supplementary Fig. S15 and the unknown concentrations of real samples were analyzed and shown in Supplementary Table S3. The obtained recovery (%) by the AAS is consistent with adopted ECL method.

## Conclusions

In conclusion, we developed a co-reactant-free solid-state ECL strategy by electrodeposition of polyluminol-gold on GCE surface and observed a stable ECL signal in O_2_ saturated neutral buffer solutions. The observed ECL intensity effectively increases with the addition of Hg^2+^ ion even at picomolar range with a good linear relationship. Even though the adopted methodology is simple and one-pot synthesis procedure involved to prepare the self-enhanced solid-state ECL platform. This is the first time we utilized a luminol-gold probe to detect Hg^2+^ with signal-on ECL platform rather than quenching. Moreover, this co-reactant-free novel solid-state self-enhanced ECL offers good recovery in the real sample analysis of Hg^2+^ ion sensing and obtained results were comparable with standard spectroscopic technique of atomic absorption spectroscope.

## Supplementary Information

Supplementary Information.
