# Supplementary material for: Co-reactant-free self-enhanced solid-state electrochemiluminescence platform based on polyluminol-gold nanocomposite for signal-on detection of mercury ion
Source: Sci Rep. 2021 Mar 25;11:6932. doi: 10.1038/s41598-021-86195-1 (PMC7994561; doi:10.1038/s41598-021-86195-1)
Supplement: Supplementary file 1 — Supplementary Information. [file 41598_2021_86195_MOESM1_ESM.doc]

Supporting Information

**Co-reactant-free self-enhanced solid-state electrochemiluminescence platform based on polyluminol-gold nanocomposite for signal-on detection of mercury ion**

Chikkili Venkateswara Raju^1,2^ and Shanmugam Senthil Kumar^1,2^*

^1^Electrodics and Electro Catalysis Division, CSIR-Central Electrochemical Research Institute (CECRI), Karaikudi - 630 003, Tamilnadu, India.

^2^Academy of Scientific and Innovative Research (AcSIR), Ghaziabad-201002, Uttar Pradesh, India.

*Corresponding author

[ssenthilmugam@gmail.com](mailto:ssenthilmugam@gmail.com); [ssenthilkumar@cecri.res.in](mailto:ssenthilkumar@cecri.res.in)


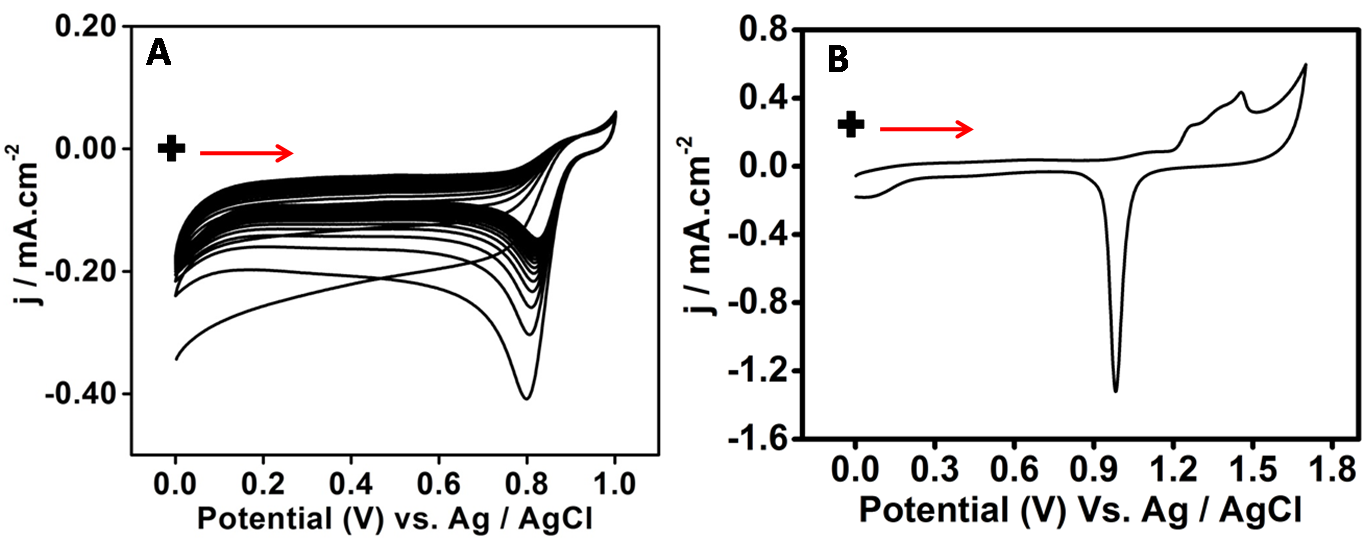


**Fig. S1.** Repetitive CVs of 1.5 mM HAuCl_4_ (A) in 0.5 M H_2_SO_4_ at the scan rate of 0.1 V/s. CV of AuNPs/GCE (B) in 0.5 M H_2_SO_4_ at a scan rate of 0.1 V/s.


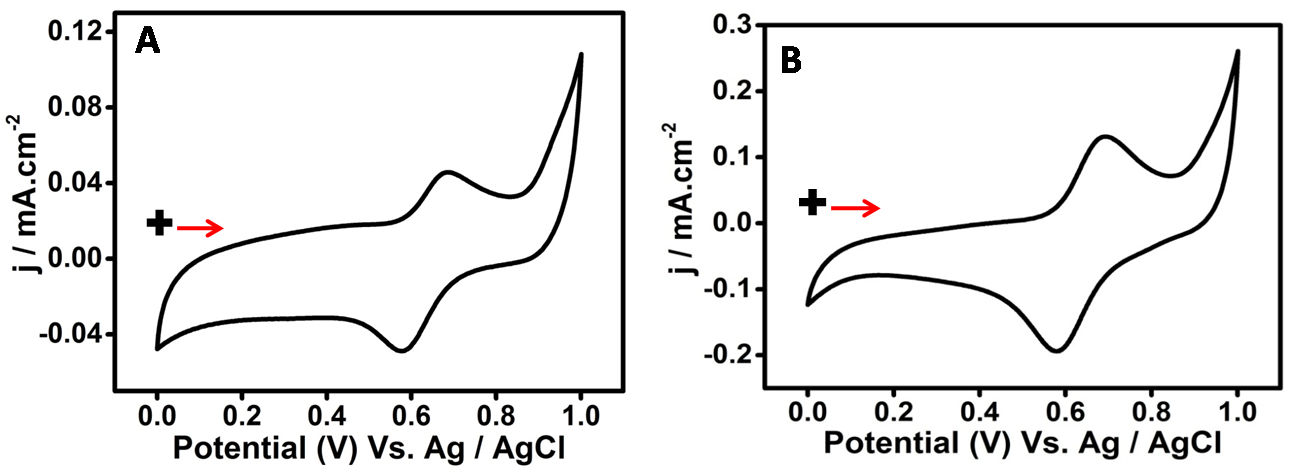
**Fig. S2.**  CV of PL/GCE (A) and (PL-Au)_nano_/GCE (B) in 0.5 M H_2_SO_4_ at the scan rate of 0.1 V/s.


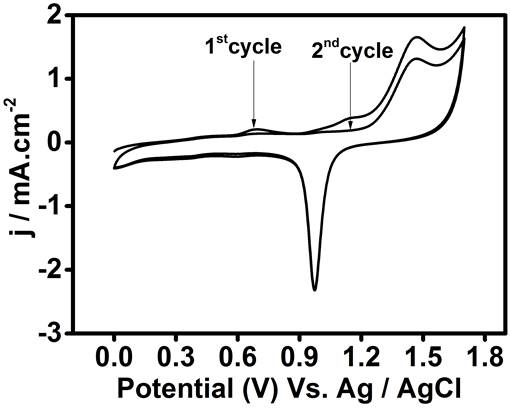


**Fig. S3.** Cyclic voltammogram of (PL-Au)_nano_/GCE in 0.5 M H_2_SO_4_ at the scan rate of 0.1 V/s.

**Table S1.** Current density (j) and Charge (Q) of PL/GCE and (PL-Au)_nano_/GCE comparison table.

| Composite | j/mA.cm^-2^  oxidation reduction | Charge (Q) in µC  oxidation reduction |
| --- | --- | --- |
| PL/GCE  (PL-Au)_nano_/GCE | 0.044 0.05  0.13 0.19 | 25 29  127 131 |

**Table S2.** The calculated charge of ORR peak at different scan directions

| S.No | Scan direction | Charge of ORR peak (Q) in µC |
| --- | --- | --- |
| 1  2  3 | -0.3 to 1 V  - 0.6 to 1 V  -0.8 to 1 V | 6  192  310 |

**1. XPS analysis**

The XPS survey spectrum of (PL-Au)_nano_/GCE was shown in Fig. S4, which shows the presence of Au, C, O and N elements, evidencing the existence of luminol along with the gold. Fig. S5 shows the high resolution XPS spectrum of (PL-Au)_nano_/GCE composite before (Fig. S5A,B) and after etching (Fig. S5C,D) of composite film. The Au binding energies were obtained at 83.78 eV and 87.48 eV (Fig. 1E,F) which corresponds to the 4f_7/2_ and 4f_5/2_ respectively ^1^. We observed two peaks for O1s at 532.1 eV and 532.9 eV (Fig. S5A) which are related to C=O and C-O-C binding energies. The N1s spectrum (Fig. S5B) also shows two peaks at 399.1 eV and 400.2 eV respectively. These peaks are due to the presence of amide nitrogen and benzenoid nitrogen respectively ^2,3^. The binding energies of O and N spectrum shows similar before and after etching but the peak intensities were changed. The peak intensities of N 1s and O 1s were decreased after the etching (Fig. S5 C, D). The EDX spectrum of PL/GCE shows carbon, nitrogen, oxygen elements which indicates the presence of lumiol, whereas (PL-Au)_nano_/GCE (Fig. S6A,B) clearly shows the Au atom along with luminol. In AFM, we observed trace of flakes like structure for PL/GCE. A well defined nano-spheres with uniform size and shape particles were observed for (PL-Au)_nano_/GCE (Fig. S6 C,D). The fine nano structure of (PL-Au)_nano_/GCE composite is due to the presence of gold atoms.


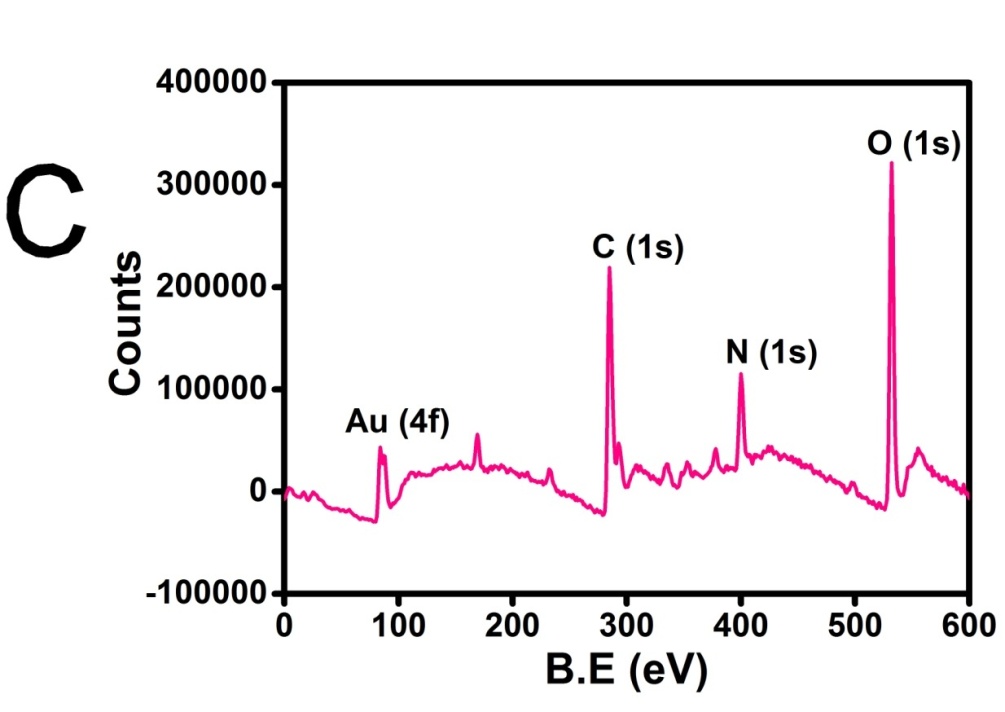


Fig. S4. XPS survey spectrum of (PL-Au)_nano_/GCE composite.


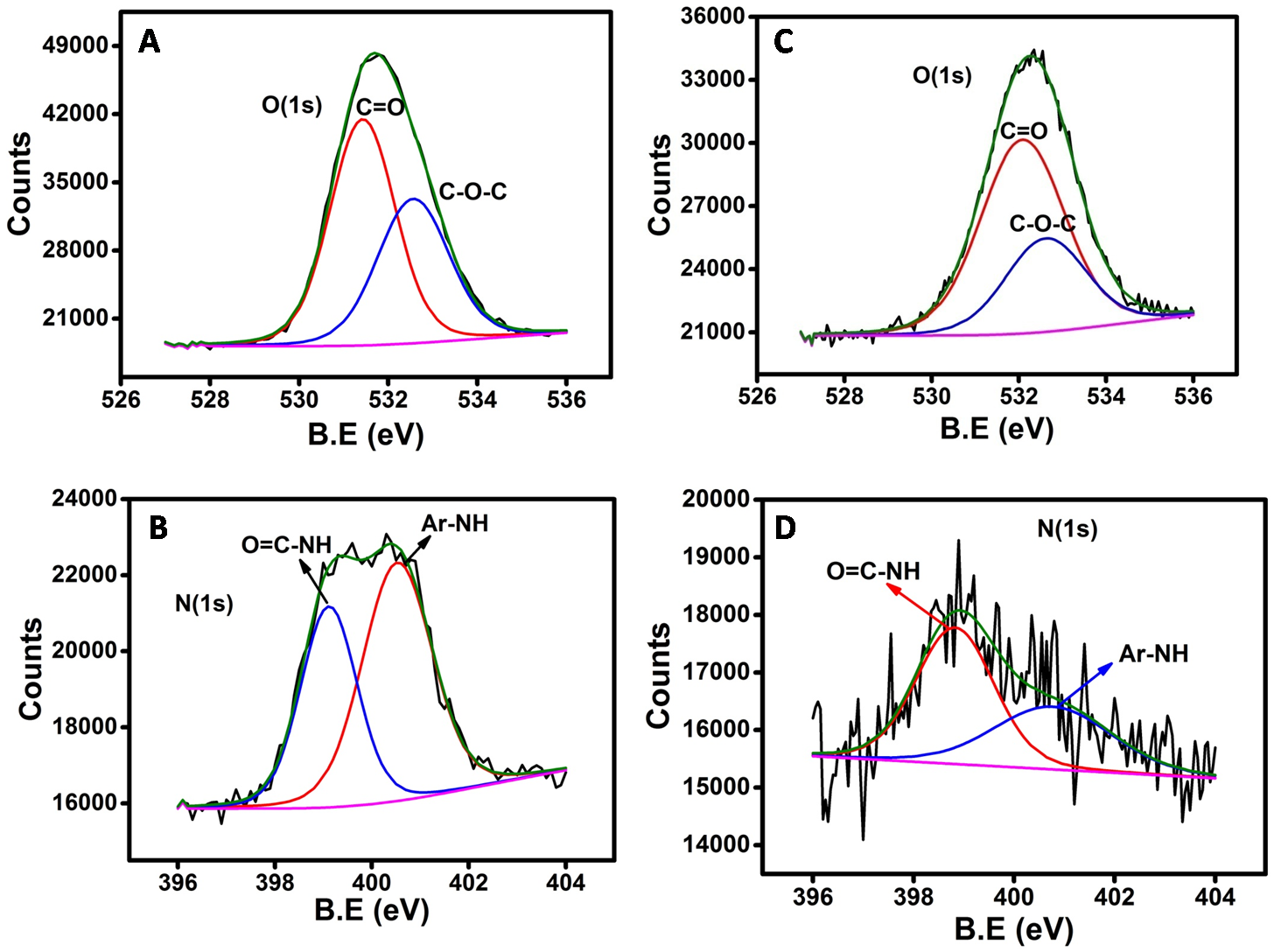


**Fig. S5.** XPS spectrum of O(1s) and N(1s) of (PL-Au)_nano_/GCE before etching (A,B) and after etching (C,D) respectively.


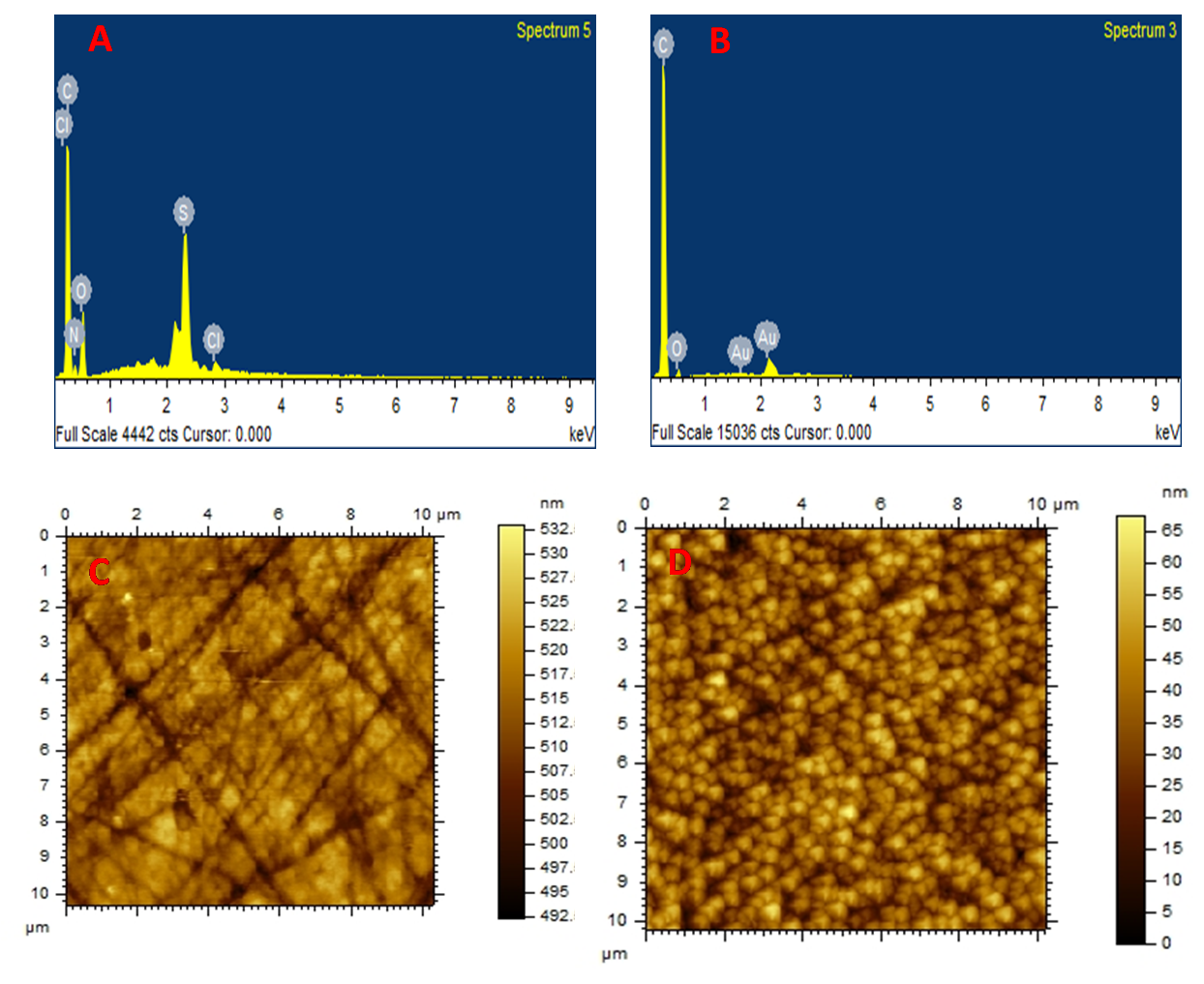


**Fig. S6.** EDX of PL/GCE (A), (PL-Au)_nano_/GCE(B), AFM images of PL/GCE (C) and (PL-Au)_nano_/GCE (D).


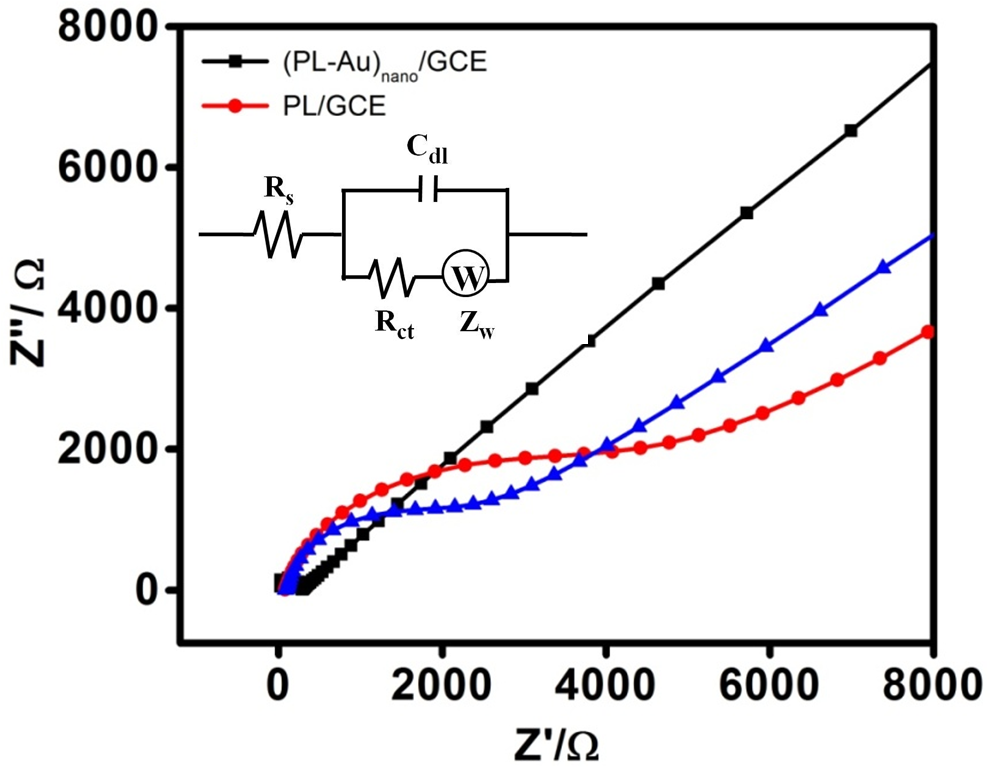


**Fig. S7.** Electrochemical impedance studies of GCE, PL/GCE and (PL-Au)_nano_/GCE in 1 mM Ferricyanide containing 0.1 M PBS. Inset is EIS circuit diagram.


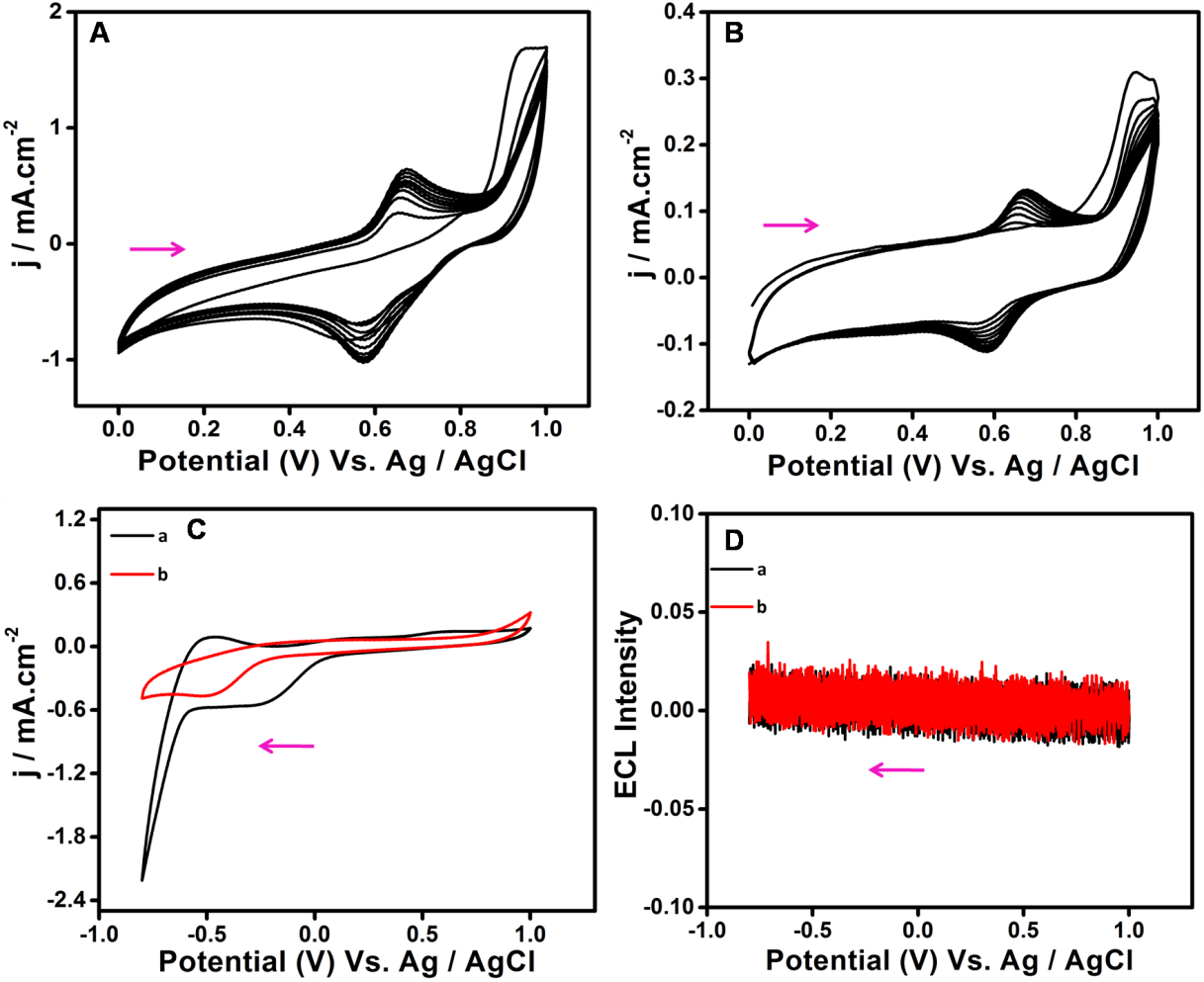


**Fig. S8.**  Repetitive CVs of 1 mM luminol+1.5 mM PtCl_4_^2-^ (A) and 1 mM luminol+1.5 mM AgNO_3_ (B) in 0.5 M H_2_SO_4_. Cyclic voltammograms (C) and its corresponding ECL signals (D) of (PL-Pt)_nano_/GCE (a), (PL-Ag)_nano_/GCE (b) in 0.1 M PBS (pH 7.4) at a scan rate of 0.1 V/s.

2. Concentration effect of luminol and HAuCl_4_.3H_2_O

We deposited (PL-Au)_nano_ on GCE surface by changing the luminol and HAuCl_4_.3H_2_O concentrations and effectively studied the ECL experiments. Initially by taking 1 mM luminol as constant we changed the HAuCl_4_.3H_2_O concentrations from 0.125 to 2 mM during the deposition (PL-Au)_nano_/GCE composite.


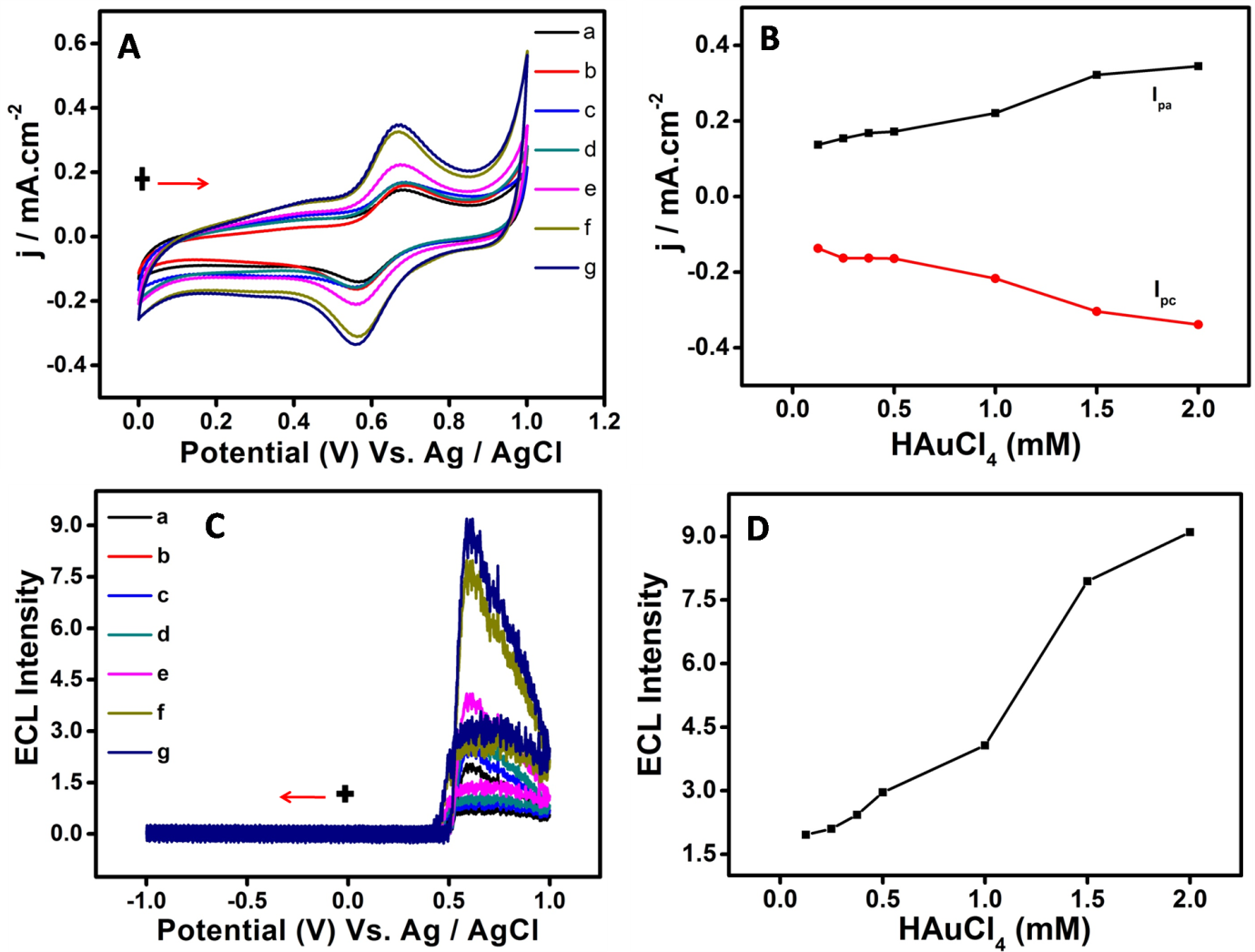


**Fig. S9.**  Cyclic voltammograms (A) of (PL-Au)_nano_/GCE after 20 cycles in 0.5 M H_2_SO_4_  after composite formation in 0.5 M H_2_SO_4_ + 0.125 (a), 0.25 (b), 0.375 (c), 0.5 (d), 1 (e), 1.5 (f) and 2 mM (g) HAuCl_4_ at a scan rate of 0.1 V/s, (B) corresponding calibration curve, (C) Corresponding ECL responses of (PL-Au)_nano_/GCE in 0.1 M PBS (pH 7.4) and (D) ECL calibration curve.

Fig. S9A indicates the CV of (PL-Au)_nano_/GCE in 0.5 M H_2_SO_4_ at 0.1 V/s at various HAuCl_4_.3H_2_O concentrations from 0.125 to 2 mM (Fig. S10A (a-g)), which is used during the deposition of composite. We find that the oxidation and reduction current of (PL-Au)_nano_/GCE increases and attains a limiting value of 1.5 mM of HAuCl_4_.3H_2_O, after that there is no change in current even at 2 mM. The derivative plot of concentration versus peak current shows in Fig. S9B. Fig. S9C is corresponding ECL signals of (PL-Au)_nano_/GCE in O_2_-saturated 0.1 M PBS (pH 7.4). As like CV, observed ECL intensity of (PL-Au)_nano_/GCE (Fig. S9C (a-g)) also increases and reaches maximum when HAuCl_4_.3H_2_O is 1.5 mM. The derivative plot for ECL intensity *vs.* concentration plot was shown in Fig. S9D. Further by keeping 1.5 mM HAuCl_4_.3H_2_O as constant we changed the luminol concentration (Fig. S10A) from 1 to 3 mM, which can be used during the cycling, and its corresponding ECL curves (Fig. S10B) in O_2_ saturated 0.1 M PBS (pH 7.4). This result reveals that there is no more change of peak current in CV and ECL signals were observed by changing luminol concentration during cycling in 0.5 M H_2_SO_4_ at 0.1 V/s.


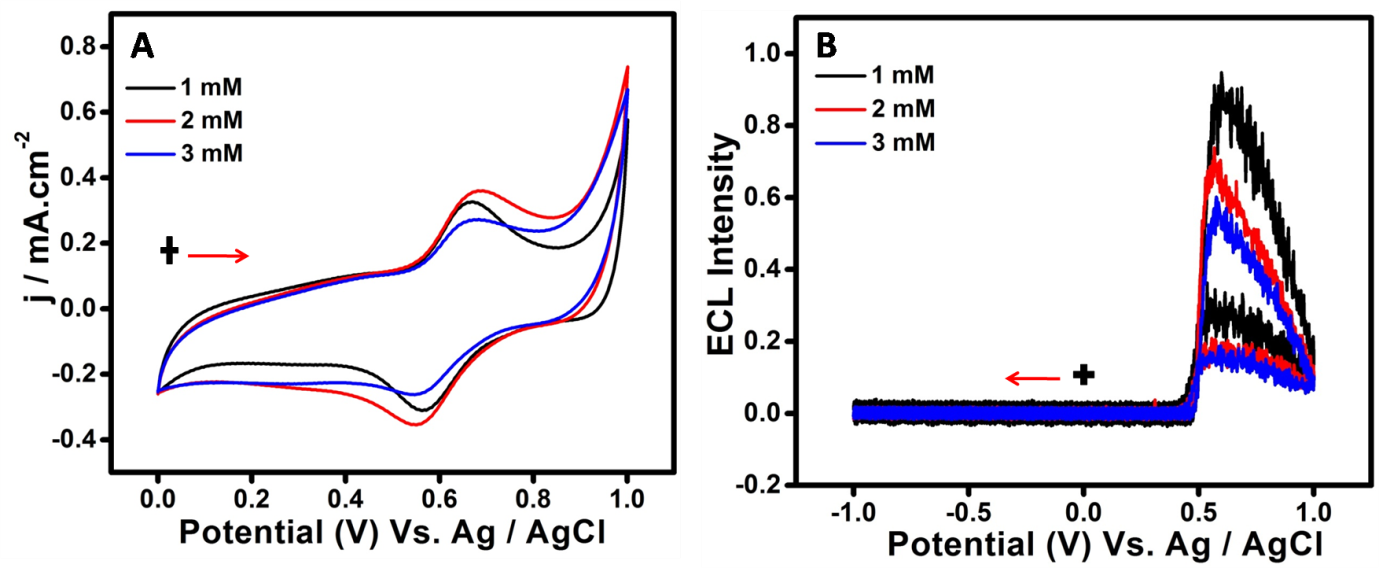


**Fig. S10.**  (A) Cyclic voltammograms of (PL-Au)_nano_/GCE in 0.5 M H_2_SO_4_ after 20 cycles in 1.5 mM HAuCl_4_ + 1 mM luminol, 1.5 mM HAuCl_4_ + 2 mM luminol and 1.5 mM HAuCl_4_ + 3 mM luminol at a scan rate of 0.1 V/s. (B) corresponding ECL in 0.1 M PBS (pH 7.4).


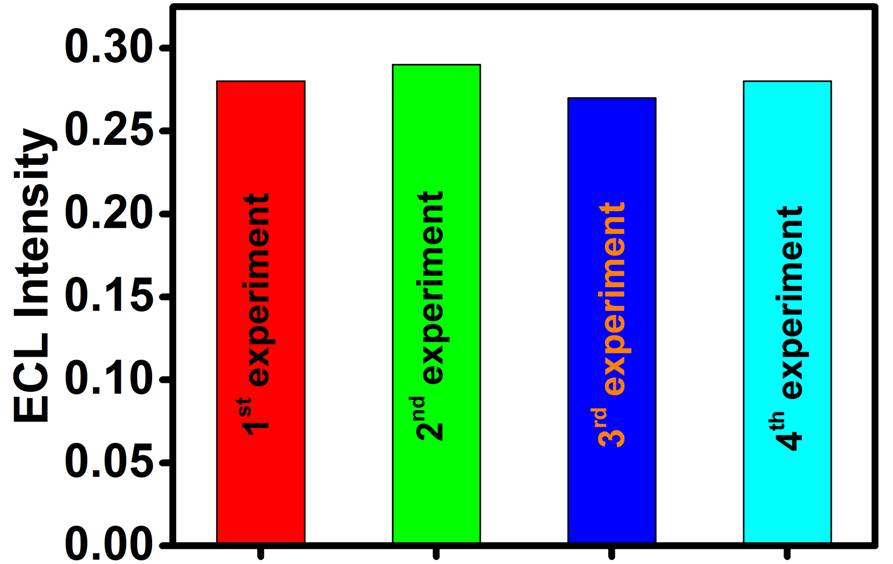


**Fig. S11.** Bar chart diagram of ECL intensity of (PL-Au)_nano_/GCE at different repetitions in O_2_ saturated 0.1 M (PBS pH 7.4).


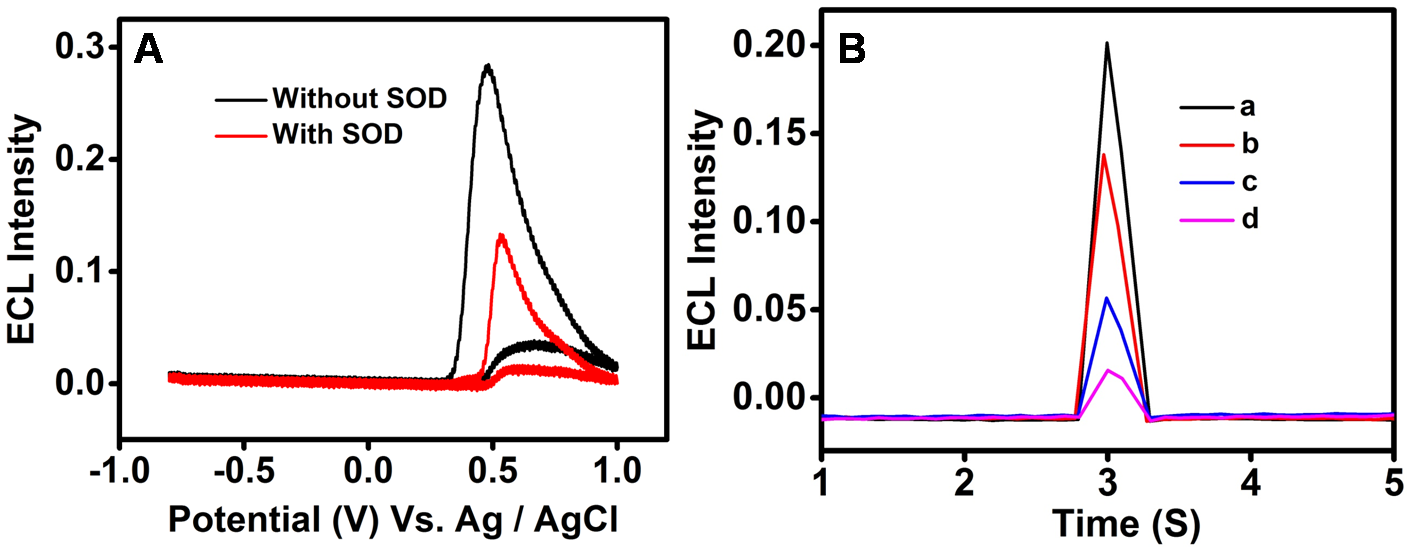


**Fig. S12.** ECL curves of (PL-Au)_nano_/GCE in the presence and absence of SOD (A). ECL intensity *vs* time (I vs t) transients of (PL-Au)_nano_/GCE at different concentrations of benzoquinone (B), 0 mM (a), 2 mM (b), 4 mM (c) and 6 mM (d) in O_2_ saturated 0.1 M PBS (pH 7.4).


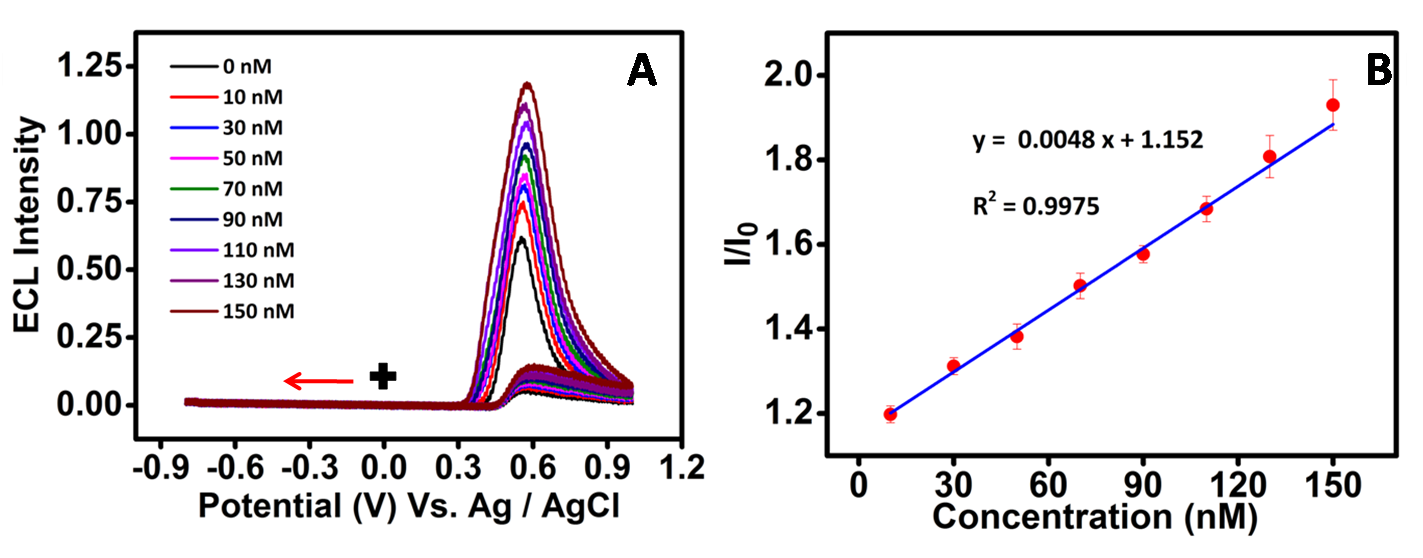


**Fig. S13.**  ECL response (A) of (PL-Au)_nano_/GCE at various Hg^2+^ concentrations 0, 10, 30, 50, 70, 90, 110, 130 and 150 nM, its calibration curve (B) in O_2_ saturated 0.1 M PBS (pH7.4) at 0.1 V/s.

**
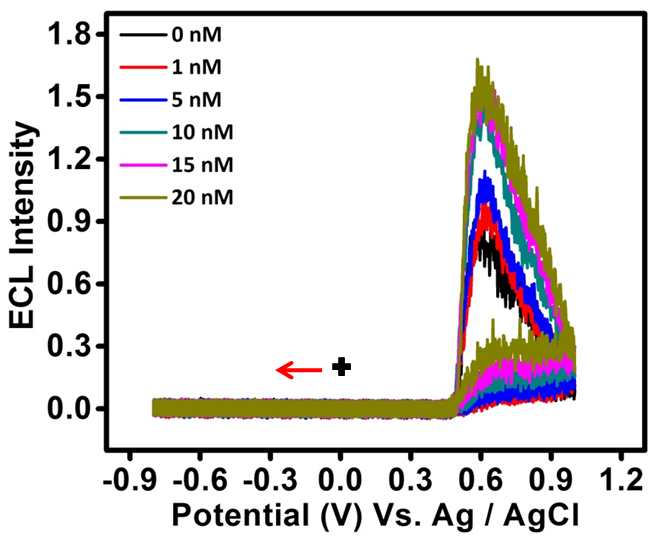
**

**Fig. S14.**  ECL signals of PL/GCE at various Hg^2+^ concentration 0 to 20 nM in 0.1 M PBS (pH 7.4).

**
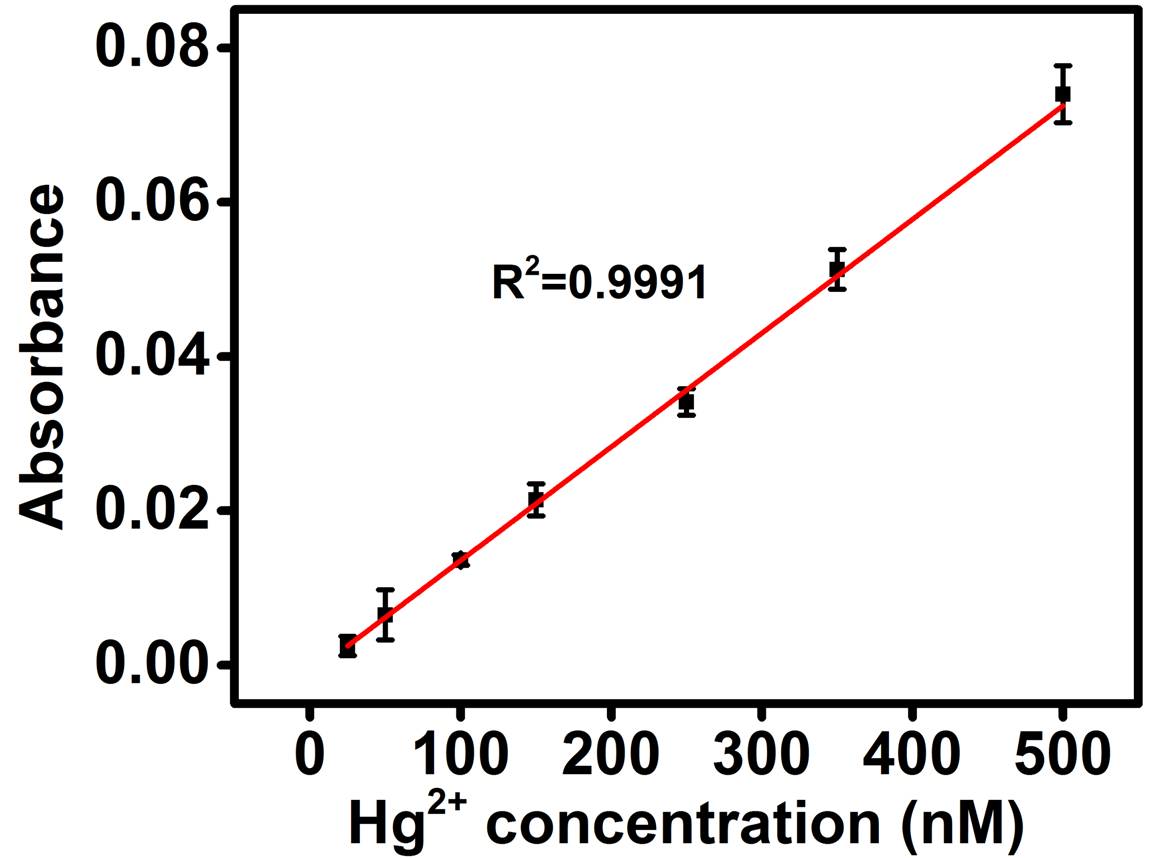
**

**Fig. S15.**  The linear plot between Hg^2+^ concentration and absorbance obtained from AAS.

| Sample | Concentration  Spiked (nM) found (nM) | Recovery (% n=3) |
| --- | --- | --- |
| Tap water  Serum | 30 28.8  50 48.8  30 30.2  50 50.2 | 96  97.6  100.6  100.4 |

**Table S3.** Hg^2+^ ion detection and recovery (%) in tap water and serum samples by using AAS technique.

3. References

1. Kumar, V. *et al.* Size-Dependent Synthesis of Gold Nanoparticles and Their Peroxidase-Like Activity for the Colorimetric Detection of Glutathione from Human Blood Serum. *ACS Sustain Chem Eng* **6**, 7662–7675 (2018).

2. Wang, C. *et al.* Facile synthesis of nitrogen-doped carbon dots from COOH-functional ionic liquid and their sensing application in selective detection of free chlorine. *Mater. Res. Express* **3**, 095020 (2016).

3. Patil, S. H., Gaikwad, A. P., Sathaye, S. D. & Patil, K. R. To form layer by layer composite film in view of its application as supercapacitor electrode by exploiting the techniques of thin films formation just around the corner. *Electrochim Acta* **265**, 556–568 (2018).
